# Poor sensitivity of "AccuPower SARS-CoV-2 real time RT-PCR kit (Bioneer, South Korea)"

**DOI:** 10.1186/s12985-020-01445-4

**Published:** 2020-11-14

**Authors:** Byron Freire-Paspuel, Miguel Angel Garcia-Bereguiain

**Affiliations:** One Health Research Group, Universidad de Las Américas, Quito, Ecuador

**Keywords:** SARS-CoV-2, RT-PCR, CDC, Bioneer

## Abstract

**Background:**

Several molecular kits are available for SARS-CoV-2 diagnosis, mostly lacking of proper clinical evaluation due to the emergency caused by COVID19 pandemia, particularly at developing countries like Ecuador.

**Objective:**

We carried out an evaluation of the clinical performance of "AccuPower SARS-CoV-2 Real Time RT-PCR kit" (Bioneer, South Korea) for SARS-CoV-2 diagnosis using 2019-nCoV CDC EUA kit (IDT, USA) as a gold standard*.*

**Results:**

48 clinical specimens were included on the study, 38 tested SARS-CoV-2 positive and 10 SARS-CoV-2 negative for 2019-nCoV CDC EUA kit. For "AccuPower SARS-CoV-2 Real Time RT-PCR kit", only 30 were SARS-CoV-2 positive, indicating a low clinical performance with sensitivity of 78.9%. Moreover, the limit of detection for "AccuPower SARS-CoV-2 Real Time RT-PCR kit" was estimated to be higher than 40,000 viral RNA copies/mL of sample.

**Conclusions:**

Proper clinical performance evaluation studies from government agencies at developing countries should be mandatory prior to clinical use authorization of SARS-CoV-2 diagnosis kits, particularly when those kits lack of either FDA or its country of origin clinical use authorization, to prevent the distribution of low quality products that may have a negative impact of COVID19 surveillance at developing countries.

## Introduction

The COVID19 outbreaks has challenged public health systems worldwide, particularly at developing countries. Not only patient cares or surveillance programs are overflow, but also the capacity for regulatory agencies to guarantee the quality of SARS-CoV-2 related diagnosis tools. For instance, multiple SARS-CoV-2 molecular diagnosis kits are available on the market, mostly based on RT-qPCR. Some of them have received emergency use authorization (EUA) from the U.S. Food and Drug Administration (FDA) [1], or at least by regulatory agencies at their country of production, while others only report clinical evaluation studies made by manufacturers.

The CDC designed 2019-nCoV CDC EUA kit (IDT, USA) is based on N1 and N2 gene targets to detect SARS-CoV-2 that have received positive evaluation on recent reports, and RNaseP target as a quality control of the RNA extraction; it is considered a gold standard for clinical evaluation worldwide [2–6].

"AccuPower SARS-CoV-2 Real Time RT-PCR kit " (Bioneer, South Korea) is a RT-qPCT kit that include two gene targets "RdRp" and "E" for SARS-CoV-2 detection, a "IPC" probe for PCR inhibition control, but no gene target for RNA extraction quality control. Although this kit lacks of EUA approval from FDA (USA) and from Korean CDC [1, 7], it has CE mark and is currently available in countries like Ecuador, Mexico and Colombia for in vitro SARS-CoV-2 clinical diagnosis.

The aim of this study was to evaluate the clinical performance in terms of sensitivity and limit of detection for "AccuPower SARS-CoV-2 Real Time RT-PCR kit " using 2019-nCoV CDC EUA kit as a gold standard for SARS-CoV-2 RT-qPCR diagnosis from nasopharyngeal samples.

## Material and methods

### Study design

48 clinical specimens (nasopharyngeal swabs collected on 0.5 mL TE pH 8 buffer) were included on this study, coming from individuals attending Universidad de Las Américas laboratory for SARS-CoV2 diagnosis in Quito (Ecuador). Also, 4 negative controls (TE pH 8 buffer) were included as control for carryover contamination, one for each set of RNA extractions.

### RNA extraction and RT-qPCR for SARS-CoV-2 diagnosis using 2019-nCoV CDC kit

All the samples included on the study were tested following a modified version of the CDC protocol: (1) using "AccuPre Viral RNA extraction kit IVD" (Bioneer, South Corea) as an alternate RNA extraction method; (2) using CFX96 BioRad instrument [2, 3, 6, 8, 9]. Final volume of RT-PCR reaction was 15 ul including 4 uL of RNA extraction.

### SARS-CoV-2 diagnosis using "AccuPower SARS-CoV-2 Real Time RT-PCR kit"

Same RNA extractions from all the samples included on the study were tested using "AccuPower SARS-CoV-2 Real Time RT-PCR kit" following manufacturer's intructions (see Additional file 1). Final volume of RT-PCR reaction was 25 µl including 5 µL of RNA extraction (for a detailed comparison among both kits see Table 3). Although RNA extraction were tested with both RT-PCR protocols within 48 h, the quality of RNA was assured by running RT-qPCR for RNaseP probe.

### Analytical sensitivity

Limit of detection (LoD) was performed using the 2019-nCoV N positive control (IDT, USA) provided at 200,000 genome equivalents/mL for 2019-nCoV CDC FDA EUA kit. As 40 µL of elution buffer volumen and 200 µL of sample are used in the RNA extraction protocol, a 200 conversion factor applied to change LoD units from copies/µL of RNA solution to copies/mL of sample. For instance, 10 copies/µL of RNA extraction are equivalent to 2000 copies/mL of sample. For "AccuPower SARS-CoV-2 Real Time RT-PCR kit ", a positive control is included on the kit but the concentration is not detailed, so it was not possible to directly determine LoD.

## Results

### Clinical performance of "AccuPower SARS-CoV-2 real time RT-PCR kit " compared to the CDC gold standard protocol

48 samples were tested for SARS-CoV-2 following both protocols described on the methods. 10 samples tested negative for either 2019-nCoV CDC EUA kit or "AccuPower SARS-CoV-2 Real Time RT-PCR kit", indicating a specificity of 100%. 38 samples tested positive for 2019-nCoV CDC EUA; from those samples, 30 samples tested positive for either E and RdRp  gene targets (23 true positives samples) or RdRp gene target only (7 inconclusive samples) for the "AccuPower SARS-CoV-2 Real Time RT-PCR kit", indicating a sensitivity of 78.9% (95% CI: 65.98–91.9%) (Tables 1 and 2). If we considered as positive samples, only true positive samples with RdRp and E gene targets amplification, the sensitivity for "AccuPower SARS-CoV-2 Real Time RT-PCR kit" would be 60.5% (95% CI: 50.7–70.6%). The quality of RNA extractions was assured by running RT-qPCR for RNaseP gene target for each RT-PCR protocol; no statistically significant differences were found for RNasaP Ct values.

**Table 1:** Clinical performance of " AccuPower SARS-CoV-2 Real Time RT-PCR kit " compared to "2019-nCoV CDC EUA kit". Value of 100% and 78.9% (95% CI: 65.98–91.9%) corresponds to specificity and sensitivity, respectively

	"AccuPower SARS-CoV-2" Positive	"AccuPower SARS-CoV-2" Negative
"2019-nCoV CDC" POSITIVE	30 (78.9%)	8
"2019-nCoV CDC" NEGATIVE	0	10 (100%)

**Table 2:** Ct values and viral loads (viral RNA copies/uL of RNA extraction solution) for samples processed with "2019-nCoV CDC EUA kit" and ""AccuPower SARS-CoV-2 kit"

Sample	Sample ID	2019-nCoV CDC EUA					Accupower SARS-CoV-2 (Bioneer)				
		Viral Load (copies/uL)	N1 Ct	N2 Ct	RP Ct	Result CDC	E Ct	IPC (M1)Ct	RdRp Ct	IPC (M2) Ct	Result Bioneer
1	13517	2.03 × 10^7^	12.16	13.13	20.16	Positive	15.38	25.95	15.38	25.67	Positive
2	11957	1.11 × 10^7^	13.08	14.04	27.02	Positive	16.10	26.31	16.19	26.14	Positive
3	13645	2.32 × 10^6^	16.15	17.21	21.28	Positive	23.25	25.44	21.69	26.10	Positive
4	11971	6.10 × 10^5^	17.50	18.78	23.35	Positive	21.31	26.06	21.29	25.92	Positive
5	13466	2.04 × 10^5^	19.17	21.31	21.01	Positive	23.06	25.52	22.27	25.81	Positive
6	13468	1.61 × 10^5^	19.53	22.02	20.23	Positive	23.47	25.94	23.50	25.99	Positive
7	12116	1.36 × 10^5^	20.57	20.66	24.29	Positive	27.01	26.78	25.26	26.07	Positive
8	12397	1.20 × 10^5^	21.61	22.25	21.04	Positive	24.28	25.93	24.11	24.93	Positive
9	13441	2.75 × 10^4^	22.70	23.62	24.34	Positive	26.26	26.00	26.06	25.65	Positive
10	11944	1.79 × 10^4^	24.02	24.69	20.54	Positive	27.20	26.09	27.55	26.03	Positive
11	13632	1.66 × 10^4^	23.26	24.60	23.83	Positive	28.12	25.62	27.93	25.89	Positive
12	12121	2.12 × 10^3^	26.87	27.39	19.30	Positive	30.23	26.03	31.12	25.65	Positive
13	13469	1.58 × 10^3^	26.58	29.64	23.00	Positive	30.34	25.82	31.27	25.75	Positive
14	12079	1.46 × 10^3^	27.23	28.06	20.15	Positive	29.60	25.99	30.23	25.74	Positive
15	12123	7.49 × 10^2^	28.41	29.09	20.07	Positive	31.60	25.90	32.98	25.68	Positive
16	12092	6.24 × 10^2^	28.68	29.24	19.36	Positive	32.06	26.04	33.23	25.98	Positive
17	13617	4.53 × 10^2^	28.66	31.31	21.41	Positive	NA	28.98	NA	26.33	Negative
18	11949	4.34 × 10^2^	28.71	30.48	22.40	Positive	33.08	26.42	33.85	26.19	Positive
19	13653	4.24 × 10^2^	28.54	30.37	19.11	Positive	31.67	25.87	33.00	25.79	Positive
20	13523	3.56 × 10^2^	28.96	31.21	17.39	Positive	31.73	26.17	32.27	26.03	Positive
21	13598	3.37 × 10^2^	29.03	31.07	19.15	Positive	32.77	25.63	34.33	25.54	Positive
22	13560	2.97 × 10^2^	29.19	30.64	19.42	Positive	NA	25.93	35.54	25.81	Inconclusive
23	12107	2.66 × 10^2^	29.94	30.92	19.65	Positive	34.02	26.23	20.16	24.55	Positive
24	11967	2.50 × 10^2^	29.39	32.14	23.86	Positive	32.74	25.81	34.08	25.79	Positive
25	9224	2.23 × 10^2^	29.47	30.94	24.89	Positive	33.16	26.06	33.20	26.13	Positive
26	9220	1.97 × 10^2^	29.65	31.07	27.88	Positive	NA	26.09	37.73	26.22	Inconclusive
27	12451	1.36 × 10^2^	30.18	32.29	23.53	Positive	NA	26.08	35.07	25.66	Inconclusive
28	13489	1.06 × 10^2^	30.49	32.88	20.17	Positive	NA	25.64	34.88	25.74	Inconclusive
29	13522	9.17 × 10^1^	30.68	34.30	18.99	Positive	NA	26.07	36.57	25.72	Inconclusive
30	13644	8.10 × 10^1^	30.92	32.81	22.91	Positive	NA	25.81	NA	25.70	Negative
31	13636	6.20 × 10^1^	31.31	34.54	23.44	Positive	NA	26.04	NA	25.67	Negative
32	11966	5.73 × 10^1^	31.63	33.47	25.19	Positive	NA	25.87	36.38	25.69	Inconclusive
33	11948	3.33 × 10^1^	31.96	35.35	22.13	Positive	NA	26.31	36.61	26.03	Inconclusive
34	13674	1.92 × 10^1^	32.65	36.06	21.26	Positive	NA	25.76	NA	25.61	Negative
35	13521	1.10 × 10^1^	34.15	36.61	21.26	Positive	NA	26.29	NA	26.03	Negative
36	11946	6.77	33.97	38.15	21.97	Positive	NA	26.02	NA	25.78	Negative
37	13616	4.45	34.50	38.68	19.66	Positive	NA	25.99	NA	25.77	Negative
38	13630	2.87	34.88	38.58	23.17	Positive	NA	25.60	NA	25.49	Negative

### Estimation of the limit of detection of "AccuPower SARS-CoV-2 Real Time RT-PCR kit"

The viral loads detailed on Table 2 were calculated running a calibration curve with 2019-nCoV N positive control (IDT, USA). The LoD for the CDC protocol was set at 1000 viral RNA copy per mL of sample (or 5 RNA copies/µL of RNA extraction solution) on previous studies [2, 6, 8–11]. Although LoD could not be calculated for "AccuPower SARS-CoV-2 Real Time RT-PCR kit" as we described on the methods, no true positive samples were obtained below 40.000 RNA copies/mL of sample (200 RNA copies/µL of RNA extraction solution) according to the CDC protocol; even the sample 13617 (Table 2) with a viral load of 453 copies/µL (90,600 copies/mL of sample) was not detected by "AccuPower SARS-CoV-2 Real Time RT-PCR kit". As the LoD is defined as the lowest viral load in which all samples are detected (100% sensitivity), our data indicates that the LoD for "AccuPower SARS-CoV-2 Real Time RT-PCR kit" is higher than 40,000 RNA copies/mL of sample, and even higher that 90,600 RNA copies/mL of sample if we considered the result for sample 13617.

A comparison among AccuPower SARS-CoV-2 Real Time RT-PCR and 2019-nCoV CDC EUA kits, including price per reaction for the Ecuadorian market, is detailed in Table 3.

**Table 3:** Comparison of 2019-nCoV CDC EUA (IDT, USA) and Accupower SARS-CoV-2 (Bioneer, South Korea) kits. Price per PCR reaction is for reagents only at Ecuadorian market values (RP target for IDT kit is for RNA extraction quality control, absent on Bioneer kit)

SARS-CoV-2 RT-PCR kit	Gene targets	Limit of detection	Price per PCR reaction
2019-nCoV CDC EUA (IDT, USA)	N1, N2 and RP	1000 viral copies/mL	7 USD
Accupower SARS-CoV-2 (Bioneer, South Korea)	E and RdRp	> 40,000 viral copies/mL	20 USD

## Discussion

The data presented on this work supports that "AccuPower SARS-CoV-2 Real Time RT-PCR kit" has a low clinical performance with at least a reduction of 21.1% on sensitivity compared to 2019-nCoV CDC FDA EUA, even up to 39.5% reduction if we do not consider the inconclusive samples with only RdRp amplification as positive. Also, the lack of any probe for RNA extraction quality control like RNaseP and the unreported concentration of positive controls provided for "AccuPower SARS-CoV-2 Real Time RT-PCR kit" that does not allow viral load calculations, are limitations to consider prior to use this kit. As we have described above, the LoD for "AccuPower SARS-CoV-2 Real Time RT-PCR kit" is estimated to be even higher than 90,600 viral copies/mL, as sample 13617 was not detected. Although the main limitation of our study is the sample size, we believe that our results are sufficient to conclude that the LoD for "AccuPower SARS-CoV-2 Real Time RT-PCR kit" is at least above 40,000 RNA copies/mL of sample. Considering the viral loads frequency distribution for SARS-CoV-2 reported to date, this high LoD would potentially exclude at least more than 20% of true positive cases if "AccuPower SARS-CoV-2 Real Time RT-PCR kit" is used for surveillance programs [12, 13].

"AccuPower SARS-CoV-2 Real Time RT-PCR kit" neither has EUA FDA approval nor Korean CDC EUA approval [1, 7], so it is not actually used for clinical diagnosis on its country of origin. However, it is available in Ecuador, where no evaluation studies were carried out by the governmental regulatory agency responsible for clinical use authorization for SARS-CoV-2 diagnosis.

## Conclusions

Worldwide high demand of reagents for SARS-CoV RT-qPCR diagnosis and supplies shortage is a fact, affecting even harder to developing countries like Ecuador. The poor sensitivity of "AccuPower SARS-CoV-2 Real Time RT-PCR kit" suggests that clinical performance studies should be mandatory to guarantee the quality of the supplies in the market for every country in the world. Our study aims to be a call for action to prevent the use of low quality SARS-CoV-2 diagnosis kits in Ecuador and other developing countries.

## Supplementary information

**Additional file 1**. Manufacturer's manual version provided with the "AccuPower SARS-CoV-2 Real Time RT-PCR" kit used on this study.

## Data Availability

All relevant data is included in the manuscript.

## Abbreviations

CDC: Center for Disease Control and Prevention, USA
EUA: Emergency Use Authorization
FDA: Food and Drug Administration
