# Supplementary material for: Poor sensitivity of "AccuPower SARS-CoV-2 real time RT-PCR kit (Bioneer, South Korea)"
Source: Virol J. 2020 Nov 14;17:178. doi: 10.1186/s12985-020-01445-4 (PMC7666399; doi:10.1186/s12985-020-01445-4)
Supplement: Supplementary file 1 — Additional file 1. Manufacturer's manual version provided with the "AccuPower SARS-CoV-2 Real Time RT-PCR" kit used on this study. [file 12985_2020_1445_MOESM1_ESM.pdf]

USER'S GUIDE

CE

Innovation • Value • Discovery

AccuPower® SARS-CoV-2 Real-Time RT-PCR Kit

-20℃

REF **SCV-2122**

IVD Qualitative test kit
SARS-CoV-2 RNA

EC REP MT Promedt Consulting GmbH Altenhofstr. 80
D-66386 St. Ingbert. Germany. Tel +49 6894-58 10 20

BIONEER
Innovation • Value • Discovery

AccuPower® SARS-CoV-2 Real-Time RT-PCR Kit

User's Guide

Σ 50

Version No.: 0.0 (2020-03-02)

Please read all the information in booklet before using the unit.

Bioneer Corporation
8-11, Munpyeongseo-ro, Daedeok-gu, Daejeon
34302, Republic of Korea
Tel: +82-1588-9788
Fax: +82-42-930-8688
Email: sales@bioneer.com
www.bioneer.com

Safety Warnings and Precautions

1) Please inquire BIONEER's Customer Service Center to obtain a copy of the Material Safety Data Sheet (MSDS) for this product.
2) Please read the User's Guide and check the integrity of all tubes, tips and other materials supplied with this kit prior to use.
3) This kit could apply with following equipment.
[Applicable Real-Time PCR machine]

- ABI 7500 Fast Real-Time PCR System, Thermo Fisher Scientific, cat.no. 4351106
- CFX96 Real-Time PCR Detection System, BIO-RAD, cat.no. 1845096
- *Exicycler*™96 Real Time PCR System, Bioneer Co., cat.no. A-2060 / A-2060-1

* For N/A extraction equipment, can use already installed extraction machine and proceed according to proper procedure.

4) Make sure that the used kit components are not confused with other lot's components.
5) This kit should be stored at -20℃ below, BIONEER guaranteed stable until the expiration date printed on the label. Please refer to product box for expiration date.
6) Please inquire BIONEER's Customer Service Center to obtain a copy of the Material Safety Data Sheet (MSDS) for this product.
7) All used disposable materials (tips and tubes contacted with samples) should handle by instructions and discard in a legally defined way.
8) Adhere to general clinical laboratory safety procedures during the experiment.

Warranty and Liability

All BIONEER products are manufactured and tested under strict quality control protocols. BIONEER guarantees the quality of all directly manufactured products until the expiration date printed on the label. If any issues are discovered relating to compromise in product quality, immediately contact BIONEER's Customer Service Center (order@bioneer.com).
BIONEER does not assume liability for misuse of the product, i.e. usage of the product for any purposes other than its intended purpose as described in the appropriate and applicable User's Guide. BIONEER assumes liability under the condition that the user discloses all information related to the problem to BIONEER in written form within 30 days of occurrence.

Legal Disclaimer

Some applications that may be performed with this kit may infringe upon existing patents in certain countries. The purchase of this kit does not include or provide a license to perform patented applications. Users may be required to obtain a license depending on country and application. BIONEER does not condone nor recommend the unlicensed use of a patented application.
The use of the kit is only for qualified and well-trained users in handling of clinical specimens and molecular biological experiments. After testing, all wastes should be processed with the fulfillment of the regulation of the country.

TECHNICAL SUPPORT& A/S

– If any issues are discovered relating to compromise in product quality and test results, immediately contact BIONEER’s Customer Service Center (ds@bioneer.com).

– For the quick and precise response, send us the information about the assay, attaching the applicable test data.

Category	Information
Nucleic Acid extraction	Nucleic acid extraction Instrument (Manufacturer / Serial No.)<br>Nucleic acid extraction reagent (Manufacturer / Lot No.)
Real–Time PCR	Real–Time PCR Instrument (Manufacturer / Serial No.)<br>Diagnosis Kit (Cat. No / Lot No.)

Trademarks

AccuPower® is a registered trademark of BIONEER Corporation, Republic of Korea.
Applied Biosystems™ is registered trademark of Thermo Fisher Scientific.
CFX96 is registered trademark of BIO–RAD.
Exicycler™ is a registered trademark of BIONEER Corporation, Republic of Korea.
FAM is a registered trademark of Applera.
Cyanine5 is a registered trademark of Detection Systmes.
Excel is a registered trademark of Microsoft.

Copyright

Copyright 2020. Bioneer Corporation. All rights reserved.

TABLE OF CONTENTS

1. INTENTED USE 1
2. INTRODUCTION 1
3. FEATURES AND PRINCIPLE OF THE TEST 2
4. CONTENTS AND RELATED INSTRUMENTS 2
   4.1. Contents of the Kit 2
   4.2. Related Instruments 2
5. STORAGE AND EXPIRATION DATE 3
6. REQUIRED MATERIALS AND EQUIPMENT (NOT PROVIDED IN THE KIT) 3
7. GENERAL PRECAUTIONS 3
8. PROTOCOL 4
   8.1. Preparation 4
   8.2. Specimen 4
   8.3. Work Flow 5
   8.4. Procedure 6
9. Troubleshooting 9
10. REFERENCE 11
11. SYMBOLS 12

1. INTENTED USE

AccuPower® SARS-CoV-2 Real-Time RT-PCR Kit is an *in vitro* diagnostic kit that helps diagnose COVID-19(Coronavirus disease 2019). This kit is designed for the detection of SARS-CoV-2 (E gene and RdRp gene) RNA from a COVID-19(Coronavirus disease-19) suspected patient's sample such as sputum, nasopharyngeal swab, oropharyngeal swab through Real-Time Polymerase chain reaction (PCR).

2. INTRODUCTION

SARS-CoV-2 is a virus that caused pneumonia in Wuhan, China, which occurred in December 2019, and is one of seven coronavirus infections in humans. It was named "2019-nCoV" in late 2019 in the sense that the human infection was first confirmed, and later changed to "SARS-CoV-2". The coronavirus of Wuhan pneumonia, which first broke out in the Chinese city of Wuhan in December 2019, was defined as a new type of coronavirus (2019-nCoV), as it was different from the existing six types of coronavirus, and was reported as the seventh human-infected coronavirus.

Following the WHO's announcement in January 2020 that the cause of the mass outbreak in Wuhan, China, has been confirmed as a new type of coronavirus, it cannot completely rule out the possibility of the disease being transmitted from person to humans to humans.
In February 2020, the WHO announced official new name of the disease caused by 2019 novel coronavirus as COVID-19
The Korea Centers for Disease Control and Prevention (KCDC) confirmed that there is a similar coronavirus and the highest homogeneity (89.1%) according to an analysis of the genetic sequence of the new corona virus released by China academia. In addition, the homogeneity with four types of coronavirus in humans was 39~43%, 50% for MERS and 77.5% for SARS. The Coronavirus is classified as a four Genus, including Alpha, Beta, Gamma, Delta, and the new coronavirus caused Wuhan pneumonia belongs to Beta genus.

Genus	Human infected coronavirus
α-1b	· HCoV-229E(1960s, Human coronavirus 229E)<br>· HCoV-NL63 (in 2004, Human coronavirus NL63)
β-2a	· HCoV-OC43(1960s, Human coronavirus OC43)<br>· HCoV-HKU1(in 2005, Human coronavirus HKU1)
β-2b	· SARS-CoV (in 2003, severe acute respiratory syndrome coronavirus)<br>· SARS-CoV-2 (in 2019, 2019 Novel Coronavirus)
β-2c	· MERS-CoV(in 2012, Middle East respiratory syndrome)

The new coronavirus goes through an incubation period of about 7 to 14 days (estimated) before developing symptoms such as fever, coughing and difficulty breathing. This may improve, but caution is required as some may cause severe pneumonia.
Therefore, if you visit Wuhan, China, the epicenter of the outbreak, you should avoid contact with local wild animals and poultry, as well as with markets at risk of infection, visits to medical institutions, fever and breathing difficulties. Currently, there are no vaccines or treatments to treat the new coronavirus.
However, depending on the patient's condition, treatment will be carried out such as inject an anti-viral drug to help withstand virus attacks and antibiotics to prevent secondary infection. To prevent the spread of the

pathogens and come up with effective measures, detection of virus in the early stages of infection is important. *AccuPower*® SARS-CoV-2 Real-Time RT-PCR Kit is provided master mix style, contained all materials required RT-PCR.

3. FEATURES AND PRINCIPLE OF THE TEST

Real-Time PCR is a technology that allows real-time monitoring of the amount of amplification products by measuring the fluorescence intensity generated by amplifying a specific DNA sequence. The amplification of the PCR product is measured using primer and probe marked with fluorescent material, which is specific to the specific sequence that is amplified, thus indicating a high specificity. TaqMan® Probe, commonly used in Real-time PCRs, is labeled as a "quencher" at the end of 5' with the fluorescent material at the end of 3' so that when combined with template DNA, the energy of the fluorescence is transferred to the quencher and does not emit fluorescence.
However, as the PCR process progresses, 5' – 3' exonuclease of DNA polymerase is activated and released fluorescence by separating it from the fluorescence probe. The fluorescence produced can be measured in real time and displayed as a PCR amplification curve at each cycle of the PCR, which enables detection of specific RNA in the specimen.
AccuPower® SARS-CoV-2 Real-Time RT-PCR Kit is master mix type, containing primers, dual-labeled fluorogenic (TaqMan®) probe, DNA polymerase, dNTPs, stabilizer and so on.

4. CONTENTS AND RELATED INSTRUMENTS

4.1. Contents of the Kit

Table 1. Contents of *AccuPower*® SARS-CoV-2 Real-Time RT-PCR Kit

Reagent		Qunatity	Unit
①	Master Mix	1,000 ㎕/tube X 1 ea	1.5ml tube (green cap)
②	Enzyme Mix	500 ㎕/tube X 1 ea	1.5ml tube (red cap)
③	Oligo Mix 1*	250 ㎕/tube X 1 ea	1.5ml tube (amber)
④	Oligo Mix 2	250 ㎕/tube X 1 ea	1.5ml tube (amber)
⑤	Positive Control (PC)	150 ㎕/tube X 1 ea	1.5ml tube (blue cap)

*** Oligo Mix 1 :There is a green band on label's right side.**

4.2. Related Instruments

AccuPower® SARS-CoV-2 Real-Time RT-PCR Kit can apply below instruments. Please refer to user's guide by instruments.

[Applicable Real-Time PCR instrument]

- ABI 7500 Fast Real-Time PCR System, Thermo Fisher Scientific, Cat. No. 4351106
- CFX96 Real-Time PCR Detection System, BIO-RAD, Cat. No. 1845096

- *Exicycler*™96 Real Time PCR System, Bioneer Inc. Co., Cat. No. A-2060 / A-2060-1

* For Nucleic acid extraction equipment, it is allowable that the extraction instrument that already installed can be used and proceeds according to proper procedure under laboratory environment condition.

5. STORAGE AND EXPIRATION DATE

AccuPower® SARS-CoV-2 Real-Time RT-PCR Kit should be stored below -20℃ away from UV/sunlight, guaranteed stable until the expiration date. Repeated freeze/thaw can affect contained master mix and enzyme mix quality, please avoid at all. If the kit is used not at once, the required quantity of components must be stored separately before use. And master mix don't store at 4℃.

6. REQUIRED MATERIALS AND EQUIPMENT (NOT PROVIDED IN THE KIT)

- Nucleic acid extraction instrument and reagent
  - For nucleic acid extraction equipment, can use already installed extraction machine and reagent and proceed according to proper procedure.
- Nucleic acid amplification instrument
  - ABI 7500 Fast Real-Time PCR System, Thermo Fisher Scientific, cat.no. 4351106
  - CFX96 Real-Time PCR Detection System, BIO-RAD, cat.no. 1845096
  - *Exicycler*™96 Real Time PCR System, Bioneer Co., cat.no. A-2060 / A-2060-1
- Appropriate disposable gloves for molecular experiment
- Appropriate adjustable volumetric micropipette set
- Sterilized filtered pipette tip
- 1.5 ㎖ micro tube or 15 ㎖ conical tube

7. GENERAL PRECAUTIONS

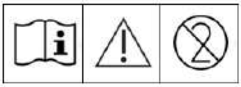

- All details follow guideline for wear personal protective equipment, collect samples, pack samples and transport samples to use this kit.
- DO NOT mix reagents from different production lots.
- Sample operations and amplification/detection operations should be performed in separate spaces.
- Do NOT omit the experimental process or make arbitrary changes to achieve the desired results.
- Perform an experiment using a sterilized filter pipette tip
- Do Not Reuse disposable materials (e.g. tips, tubes, etc. in contact with samples) used in the experiment and it should be handled and disposed according to the instructions and national guideline.
- All samples and PC RNA should be store in a freezer separately from other reagents.
- All kit components should be allowed to slowly thaw for at least 10 minutes.
- Prepare thawed kit components for best results by vortexing and spin-down prior to use.
- All positive controls (PC) should be added in a physically separate location from where the reaction mixture is reconstituted.

8. PROTOCOL

8.1. Preparation

We recommend that several precautionary measures be taken for the safety of user and laboratory, and also for the prevention of laboratory environmental contamination.

8.1.1. Proper Use of a Biosafety Cabinet (BSC)

When handling clinical samples, all related works (i.e. decapping, pipetting, capping of clinical samples and containers) should be conducted within a negative pressure biosafety cabinet (class Ⅱ or III). Negative pressure biosafety cabinet sends air from the laboratory space outside. In other words, air flows inward. This airflow prevents dangerous substances from contaminating the laboratory environment.

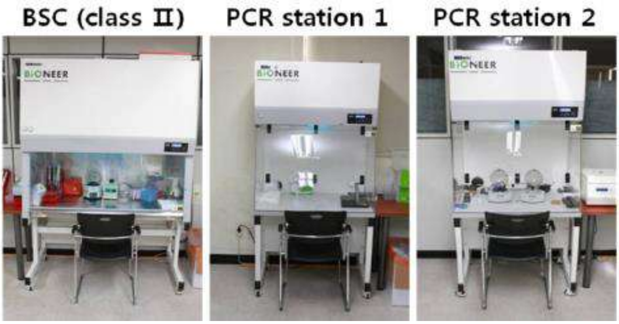

Handling clinicals specimens	Preparation of nucleic acid preparation kit contents	After the nucleic acid preparation (Sealing and vertexing the PCR tube.

Fig. 1 Biosafety Cabinet (BSC)

8.2. Specimen

⚠ All clinical samples could be treated as infectious substance.
Recommended specimens are respiratory specimens such as sputum, nasopharyngeal swab, oropharyngeal swab.

8.2.1. Specimen Collection

AccuPower® SARS-CoV-2 Real-Time RT-PCR Kit is optimized for viral RNA extracted from specimen. All samples should be kept in preservative-free containers.

8.2.2. Specimen Transport

All samples should be transported in a shatterproof transport container to prevent potential infection from sample leakage. In addition, transportation it at 4˚C and it is recommended to keep it at -70˚C if it is not possible to transport it within 48 hours samples should be transported according to local/national guidelines regarding biohazard transportation.

8.2.3. Specimen Storage

The detailed specimen storages follow the local/national guidelines such as US CDC guidelines (Interim Guideline for Collecting, Handling, and Testing Clinical Specimens from Persons Under Investigation for 2019 Novel Coronavirus, Feb, 2, 2020). For longer period of storage, samples should be stored -20˚C to -80˚C in aliquots to avoid repeated freeze/thaw cycles.

8.2.4. Interfering Substances

Clinical samples, such as nasopharyngeal, oropharyngeal swab and sputum, may contain substances which interfere with PCR. For efficient PCR, such inhibitors must be removed during the RNA extraction and purification process.

8.3. Work Flow

AccuPower® SARS-CoV-2 Real-Time RT-PCR Kits are available on the equipment specified in 4.2. Schematic workflow is shown in Fig. 2.

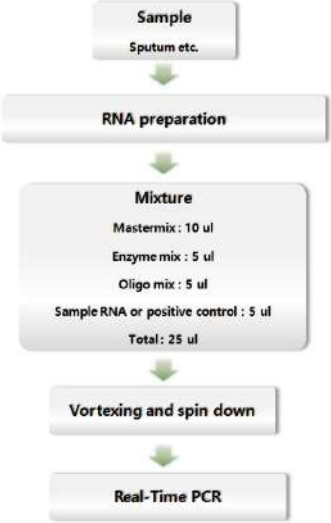

Fig. 2 Work flow

8.4. Procedure

8.4.1. Nucleic Acid Extraction

SARS-CoV-2 RNA should be purified from clinical samples. Use a viral RNA extraction kit accordingly following the manufacturer's instructions.

Note: The extraction protocol or extraction kit's quality may affect real-time PCR results.
Note: Clinical samples may contain several inhibitors, which affects PCR results. We recommend the proper extraction protocol that can remove PCR inhibitors during the extraction process.

8.4.2. PCR Preparation

1) The appropriate number of reagents and extracted nucleic acids (if frozen) should be thawed for at least 10 minutes at room temperature.
   Note: Use at least one negative control (RNA free water) and one positive control per a test as experimental controls.
   Tube 1 (E gene): 2 (experimental controls) + number of samples
   Tube 2 (RdRp gene): 2 (experimental controls) + number of samples

2) Vortex more than 10 seconds and briefly spin-down all reagents, controls and samples prior to use
   Note: Before use Master Mix, Enzyme Mix and Oligo Mix, place in the ice tray to keep the temperature. Master Mix, Enzyme Mix

3) Make a reaction mixture. Make the mixture for at least one more tube than is actually needed to account for tube adhesion loss of the mixture. Vortex and spin-down the mixture for more than 10 seconds before use. Below table2 is an example for preparation of reaction mixture.

Table 2. Reaction mixture

Number of samples (+extra)	1	8 (+2)	16 (+4)
Master Mix	10 ㎕	100 ㎕	200 ㎕
Enzyme Mix	5 ㎕	50 ㎕	100 ㎕
Oligo Mix (each)	5 ㎕	50 ㎕	100 ㎕
Total volume	20 ㎕	200 ㎕	400 ㎕
Volume per well	20 ㎕	Each 20 ㎕	Each 20 ㎕

Note: Since the oligo mix is divided into two parts, two tubes should be used per a sample. When making a reaction mixture, the tube to use Oligo Mix No.1 is labeled as 'Tube 1', and the Oligo Mix No. 2 is labeled as 'Tube 2'.

4) Load 20 ㎕ of reaction mixture made in step 3 into all PCR tube.

Target	Tube No.	Note
E gene	1	Oligo Mix 1
RdRp gene	2	Oligo Mix 2

5) Add 5 ㎕ of RNase free water as NTC into the tubes. Please seal the tubes to prevent contamination from subsequent steps.

6) Move the tubes to a separated location before proceeding to prevent contamination. Add 5 ㎕ of PC to the tubes.

7) Add 5 ㎕ of nucleic acid extracts to the remaining tubes, making a note of which samples correspond to which original clinical sample.

8) To mix the reagent with RNA sample, conduct the vortex and spin-down.

9) After Spin-down, set the PCR protocol in the PCR instrument. PCR protocol.
Note: Reporter according to target is shown below table
Note: Program setting of PCR instrument should follow the instructions of the equipment manufacturer.

Table 3. Reporter dye setting

Target	Reporter
E gene	FAM
RdRp gene	FAM
IPC	Cyanine 5

Table 4. PCR protocol

Step	Temperature (℃)	Time	Cycle
Reverse transcription	50	30min	1
Pre-denaturation	94	10min	1
Denaturation	95	15sec	45
Annealing & extension	60	1min	
Scan			

10) Place the PCR tube into the PCR instrument and perform the test.

8.4.3. Data Analysis

1) The data is analyzed according to the analysis program of each instrument manufacturer.
   Note: If use the ABI 7500 Fast, set the threshold is 0.05.
   Note: If use the CFX96, set the threshold is 200.
   Note: For *Exicycler*™96 (cat.no. A-2060 / A-2060-1), additional setting is not needed as it is automatically analyzed.
2) The analyzed Ct value of the SARS-CoV-2 Real-Time RT-PCR kit is displayed in the analysis program.
   - **Well**: Displays the locations of PC, IPC, and samples in the 96-well thermal block.
   - **Sample ID**: Displays the sample information, NTC and PC.
   - **NTC:** In every test, one well of NTC is included. And This is an indicator that checks for contamination caused by sample dispensing, nucleic acid extraction, and PCR preparation to prevent false positive results and to verify the validity of the test.
   - **PC** : Whenever a test is in progress, the PC is used to validate the test. If the Ct value of the target generated from the PC falls within the valid range, the materials and test are validated.
   - **IPC**: To check whether PCR is inhibited by the sample and to determine the amplification of nucleic acids in each well. High concentrations of target RNA can lead to reduced or absent fluorescence signal of IPC signal due to PCR competition. The validity of IPC is determined by Ct value of IPC signal. If its Ct value is within the specified range, it is valid.
   - **Target Ct**: Displays the Ct value for each target for each well. **Ct of NTC must be $\geq$ 40** Ct or negative and Ct value of PC well must be within the validity range..
   - **Target Result**: The result of the sample is reported as a Ct value. Based on PC, IPC, and sample results, the sample results are finally determined. The results are shown in the following table.
   - **Interpretation:**

   ● **ABI 7500 Fast and CFX 96:**

   If **the Ct value of the E gene and the RdRp gene** is $\leq$ **38**, it is determined as "**Positive**", and if **the Ct value is > 38**, it is determined as "**Negative**".

   ● ***Exicycler*™ 96:**

   If **the Ct value of the E gene and the RdRp gene** is $\leq$ **39**, it is determined as "**Positive**", and if **the Ct value is > 39**, it is determined as "**Negative**".

Table 5. Result analysis

Case	Result			Interpretation
	E gene (tube 1)	RdRp gene (tube 2)	IPC	
1	+	-	+	Coronavirus
2	+	+	+	SARS-CoV-2
3	-	-	+	Negative
4	-	+	+	Re-test
5	+	+	-	
6	+	-	-	
7	-	+	-	

9. Troubleshooting

Table 6. Comments and suggestions

Internal Positive Control (IPC) invalid results	
If the Cyanine5 (IPC) fluorescence signal was not detected in all wells (including controls).	• Extraction and/or PCR configuration error<br>☞Make sure that the correct extraction/PCR protocol was programmed and performed in accordance with the Kits. Repeat the assay, if necessary.<br>See User's Guide 8. PROTOCOL<br><br>• Incorrect extraction or PCR kit use<br>☞ Make sure that you use proper kits for the intended tests.<br><br>• The kit may have spoiled, due to bad storage or expiration<br>☞ Assess your storage conditions and review the expiration date. Repeat the assay with new reagents, if necessary.<br>See User's Guide 5. STORAGE AND EXPIRATION DATE
If the Cyanine5 (IPC) fluorescence signal was not detected in particular wells.	• Inhibition of PCR<br>☞ Clinical samples may contain a variety of PCR inhibitors. Repeat the assay from the sample pretreatment process which can reduce PCR inhibition.<br>☞Make sure that you use the validated sample pretreatment method in accordance with the sample type.

PC invalid results	
If the PC fluorescence signal (FAM) was undetermined.	• The kit may have spoiled, due to bad storage or expiration.<br>☞ Assess your storage conditions and review the expiration date. Repeat the assay with new reagents, if necessary.<br>See User's Guide 5. STORAGE AND EXPIRATION DATE.<br><br>• Re-use of reagents<br>☞Make sure not to re-use reagents. Re-use or repeated freeze/thaw cycles of reagents may affect the kit quality and the results of assay conclusively. Repeat the assay with new reagents, if necessary.<br>See User's Guide 5. STORAGE AND EXPIRATION DATE, 7. GENERAL PRECAUTIONS.<br><br>• PCR Protocol error<br>☞ Review your reaction preparation procedure. Confirm the amount of PC used in a single well.<br>See User's Guide 8.4.2 PCR Preparation.<br>• There may have been a pipetting error.<br>☞ Review the pipetting technique and calibration.
**No template Control (NTC) invalid results**	
If the target fluorescence signal (FAM) was detected in NTC well.	• Contamination may have occurred.<br>☞ Make sure that work space and instruments are decontaminated and repeat the assay.<br>• The kit may have spoiled, due to bad storage or expiration.<br>☞ Assess your storage conditions and review the expiration date. Repeat the assay with new reagents, if necessary.<br>See User's Guide 5. STORAGE AND EXPIRATION DATE.<br>• PCR Protocol error<br>☞ Review your reaction preparation procedure. Confirm whether controls and samples are loaded in proper wells which are assigned through S/W protocol (especially NTC well(s)).<br>See User's Guide 8.4.2 PCR Preparation.<br>• There may have been a pipetting error.<br>☞ Review the pipetting technique and calibration.

10. REFERENCE

Mackay IM. (2004) Real-time PCR in the microbiology laboratory. Clin. Microbiol. Infect. 10:190-212

NCCLS. Protocols for Determination of Limits of Detection and Limits of Quantitation; Approved Guideline. NCCLS document EP17-A

A new coronavirus pneumonia occurred in Hubei, China. (KCDC, Korea Centers for Disease Control and Prevention)

New coronavirus infection situation (2019~2020)

New coronavirus infection_procedure(For local government) (4th edition)

V M Corman et al. (2012) Detection of a novel human coronavirus by real-time reverse-transcription polymerase chain reaction. Eurosurveillance Edition. Vol.17, Issue 39

MERS response guideline (4thedition) (2016) KCDC, Korea Centers for Disease Control and Prevention

11. SYMBOLS

Symbol	Meaning	Symbol	Meaning
REF	Catalog number		Temperature limitation
IVD	*In vitro* diagnostic medical device	Σ	Contains sufficient for test
	Manufacturer	!	Caution, consult accompanying documents
LOT	Batch code		Expiration date
	Do not reuse	i	Consult instructions for use
CE	Conformite Europeenne Mark	EC REP	Authorized representative in the European Community

Bioneer Worldwide

Bioneer Corporation

Address	8-11 Munpyeongseo-ro, Daedeok-gu, Daejeon, 34302, Republic of Korea
Tel	+82-42-930-8777 (Korea: 1588-9788)
Fax	+82-42-930-8688
E-mail	sales@bioneer.com
Web	www.bioneer.com

Bioneer Inc.

Address	155 Filbert St. Suite 216 Oakland, CA 94607, USA
Tel	+1-877-264-4300 (Toll-free)
Fax	+1-510-865-0350
E-mail	order.usa@bioneer.com
Web	us.bioneer.com

Bioneer R&D Center

Address	Korea Bio Park BLDG #B-702, 700 Daewangpangyo-ro, Bundang-gu, Seongnam-si Gyeonggi-do, 13488, Republic of Korea
Tel	+82-31-628-0500
Fax	+82-31-628-0555
E-mail	sales@bioneer.co.kr
Web	www.bioneer.co.kr
